# Gluten-Induced Extra-Intestinal Manifestations in Potential Celiac Disease—Celiac Trait

**DOI:** 10.3390/nu11020320

**Published:** 2019-02-01

**Authors:** Alina Popp, Markku Mäki

**Affiliations:** 1 University of Medicine and Pharmacy “Carol Davila” and National Institute for Mother and Child Health “Alessandrescu-Rusescu”, Bucharest 020395, Romania; alina.popp@uta.fi; 2 Faculty of Medicine and Health Technology, Tampere University and Tampere University Hospital, 33520 Tampere, Finland

**Keywords:** gluten, latent celiac disease, potential celiac disease, extra-intestinal manifestations, mild enteropathy, early developing celiac disease, genetic gluten intolerance, natural history, celiac trait

## Abstract

Celiac disease patients may suffer from a number of extra-intestinal diseases related to long-term gluten ingestion. The diagnosis of celiac disease is based on the presence of a manifest small intestinal mucosal lesion. Individuals with a normal biopsy but an increased risk of developing celiac disease are referred to as potential celiac disease patients. However, these patients are not treated. This review highlights that patients with normal biopsies may suffer from the same extra-intestinal gluten-induced complications before the disease manifests at the intestinal level. We discuss diagnostic markers revealing true potential celiac disease. The evidence-based medical literature shows that these potential patients, who are “excluded” for celiac disease would in fact benefit from gluten-free diets. The question is why wait for an end-stage disease to occur when it can be prevented? We utilize research on dermatitis herpetiformis, which is a model disease in which a gluten-induced entity erupts in the skin irrespective of the state of the small intestinal mucosal morphology. Furthermore, gluten ataxia can be categorized as its own entity. The other extra-intestinal manifestations occurring in celiac disease are also found at the latent disease stage. Consequently, patients with celiac traits should be identified and treated.

## 1. Introduction

Celiac disease is an autoimmune systemic disorder in genetically susceptible persons perpetuated by the daily ingestion of gluten cereals (wheat, rye, and barley) with manifestations in the small intestine and organs outside the gut. Patients diagnosed with celiac disease show gluten-induced and gluten-dependent duodenal mucosal lesions (i.e., the typical crypt hyperplastic lesion with villous atrophy). Clinically these newly diagnosed patients may or may not be suffering from gastrointestinal symptoms. A gluten-driven extra-intestinal manifestation is often the only clue for the disease. In the primary care and within different medical disciplines, physicians should suspect celiac disease and perform case finding by serum autoantibody screening. Positive serology is often the only way to identify potential patients for a diagnostic upper intestinal endoscopy [1,2,3]. In fact, less than half of all adult patients diagnosed with celiac disease complain of gastrointestinal symptoms at an initial diagnosis [4]. This knowledge comes from Finland, where adult celiac disease diagnoses have increased 20 times in recent decades and 0.8% of the total population has a biopsy-confirmed diagnosis [5,6,7].

Patients diagnosed with celiac disease including a duodenal mucosal lesion may suffer from a number of extra-intestinal diseases [2,3]. Dermatitis herpetiformis manifests outside the gut, is gluten driven and dependent [8,9], has the same genetic background, and occurs within the same families as celiac disease [9,10]. In fact, one identical twin may have celiac disease while the other suffers from dermatitis herpetiformis [11]. Other gluten-driven extra-intestinal manifestations in celiac disease include osteopenia, osteoporosis, fractures [12,13,14], permanent tooth enamel defects [6,15], arthritis, and arthralgia [16,17,18] as well as further central and peripheral nervous system [19,20,21,22], liver [23,24,25], and reproductive system involvements [26,27]. Even autoimmune diseases may be gluten driven [28,29], and there is a risk for malignant complications, especially non-Hodgkin lymphoma, in untreated celiac disease [30,31].

By definition, celiac disease is excluded in patients who have normal small intestinal mucosal morphology at their first diagnostic endoscopy if they have been following a normal gluten-containing diet. However, it seems evident that this is not accurate. In a review in 2001, we wrote that gluten-induced extra-intestinal manifestations may develop at the latent disease stage when the mucosa is still morphologically normal [32], citing several observations [19,33,34,35,36]. Today such patients are often referred to as having “potential celiac disease” because they are found to be “normal” on biopsy [37]. Meanwhile, dermatitis herpetiformis is our model disease, in which an extra-intestinal manifestation is treated with a gluten-free diet irrespective of the mucosal finding (diseased or not). We reviewed the literature for evidence for extra-intestinal gluten-dependent manifestations in patients “excluded” for celiac disease. In this paper, we also discuss tools for identifying these “potential” treatable patients.

## 2. Latent or Potential Celiac Disease

The “pre-celiac” state has been described in patients with dermatitis herpetiformis, in whom small intestinal mucosal deterioration was shown to occur after adding extra gluten to the diet [38,39,40]. An extra gluten load also induced mucosal lesions in healthy individuals [39,41]. The concept of latent celiac disease, without having an extra load of gluten, was shown to be part of the gluten sensitivity spectrum and natural history of celiac disease [42,43,44,45]. Specifically, this was shown in Finland in celiac patients who, by chance, had previously undergone a small intestinal biopsy that was reported as normal or who were followed up because of positive serum autoantibody results.

We chose to use the term “latent celiac disease”, which is similar to Weinstein [38], when referring to patients with a normal biopsy who later exhibited mucosal deterioration and were diagnosed as celiac disease patients. For us, “latent” means existing but not manifest (i.e., the disease exists but is not manifest at the mucosal level). Based on our early descriptions, Ferguson et al. (1993) defined latent celiac disease as follows: “This term should only be applied to patients who fulfill the following conditions: (i) have a normal jejunal biopsy while taking a normal diet, (ii) at some other time, before or since, have had a flat jejunal biopsy which recovers on a gluten free diet” [46]. Following the first reports on existing celiac disease latency, a numbers of other results have been published, with early papers by Troncone [47] and Corazza et al. [48]. It is now clear that oral tolerance towards gluten can be kept for longer periods, even for decades and into older age [49].

The term “potential celiac disease” has been used interchangeably with latent celiac disease, and this has led to confusion in the celiac disease literature. Thus, the Oslo task force discouraged the use of the term “latent celiac disease”, and individuals with a normal outcome from a small intestinal biopsy but who are at increased risk of developing celiac disease based on positive celiac disease serology should be referred to as having potential celiac disease [37]. These “potential patients” are not treated as celiac. The question is, what should a gluten-triggered and gluten-dependent treatable disease outside the gut be called, when the extra-intestinal manifestation occurs at the latent stage of the disease and when conventional diagnostic biopsy criteria have excluded celiac disease? We infer that such patients should not be categorized as having potential celiac disease. Rather, they require proper treatment [50,51].

**Figure 1:**
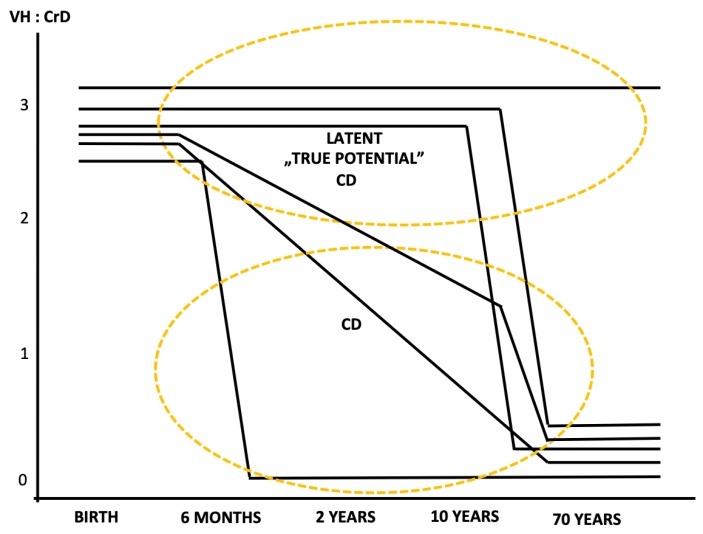
Natural history of developing celiac disease (CD) at the small intestinal mucosal level. Each line represents one individual. We are born with a normal mucosal morphology, a villus height (VH), and a crypt depth (CrD) ratio of approximately three and villi three times taller than crypts are deep. Upon gluten ingestion, mucosal injury proceeds rapidly or gradually at different ages, in childhood or only at an older age. Before developing a manifest mucosal lesion (diseased mucosa on biopsy, VH:CrD <2) every CD patient belongs to the category latent “true potential” CD (normal on biopsy, VH:CrD >2).

In Figure 1, we summarize the lifespan natural history of celiac gluten sensitivity, where each line represents a single individual. The term celiac gluten sensitivity encompasses celiac traits, latent celiac disease, genetic gluten intolerance, mild enteropathy celiac disease, early developing celiac disease, and celiac disease itself [1,32,50,51,52,53]. The gluten-induced mucosal damage develops rapidly or gradually from normal mucosal morphology to a manifest mucosal lesion. The tolerance towards gluten is individual, and it may be broken after only months or years (childhood celiac disease) but also at adolescence, adulthood, and even after decades of gluten ingestion in old age. The latent celiac disease patients, the “true potential” patients in Figure 1 (i.e., normal on biopsy showing villus height crypt depth ratio >2), are classified as having celiac disease only when the disease has deteriorated to the degree of a manifest mucosal lesion (i.e., villus height crypt depth ratio <2).

## 3. Markers of Existing Early Disease

An existing gluten-dependent disease without evidence of enteropathy until a later age - latent celiac disease - includes in its definition the susceptibility genes for celiac disease and the genes encoding the ***human leukocyte antigen*** (HLA) DQ2 or DQ8 molecules [1,32,54]. This is a check that clinicians may perform when there are symptoms and signs suggestive of celiac disease, but the biopsy is normal or does not show clear crypt hyperplasia. It shows only inflammation and descriptive mild villus atrophy. Positivity for DQ2 or DQ8 does not mean very much, since 30% to 40% of the citizens in the country are positive, but double negative means that no celiac disease will develop.

Patients positive for ***celiac disease serology*** with normal biopsies should be considered to have potential celiac disease [37]. However, gliadin antibody positivity, which is a frequent finding in celiac disease control patients and even in healthy individuals, does not correlate with celiac disease susceptibility genes [55]. Currently, tissue transglutaminase autoantibody (TG2-ab) testing is used to screen for celiac disease. It should be noted, however, that not all serum TG2-abs predict celiac disease [56]. TG2-abs have been described in other autoimmune diseases as well as in infections, tumors, myocardial damage, liver disorders, and psoriasis [54]. These antibodies are not associated with endomysial autoantibodies and may occur in persons negative both for HLA DQ2 and DQ8. The serum endomysial antibody test is the gold standard, and the presence of these autoantibodies predicts impending celiac disease [1,45,50,53,55]. In celiac disease in patients with extra-intestinal manifestations, other autoantibodies play a role in diagnosis and potentially in disease mechanisms [57].

**Figure 2:**
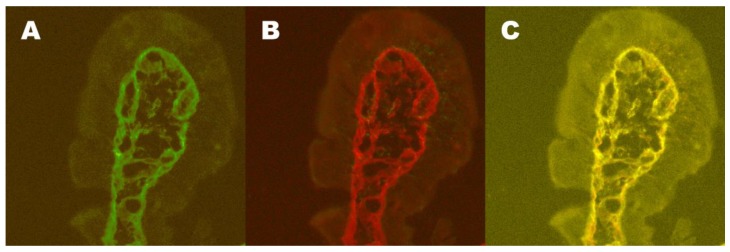
Small intestinal mucosal immunoglobulin (Ig) A deposits are shown in a villus tip from a dermatitis herpetiformis patient with normal mucosal morphology. IgA is stained with green (**A**), transglutaminase 2 (TG2) with red (**B**), and subepithelial colocalisation of IgA and TG2 can be seen in yellow (**C**).

At the mucosal level, inflammation, as measured as the density of intraepithelial T cells (***IELs***), is a very unspecific finding, but is also gluten-dependent in cases of celiac disease [58]. Marsh 1 lesions with increased IELs were shown to have a sensitivity of 59% and specificity of 57% in predicting forthcoming celiac disease [59]. However, when searched for, an autoimmune insult to the morphologically normal intestinal mucosa is, in fact, present. A high density of ***γδ T-cell-receptor-bearing IELs*** in patients with morphologically normal mucosa who also carry the susceptibility genes for celiac disease seems to be a prerequisite for developing celiac disease [43,60,61]. Yet, even if an increased density of γδ T cells is found in latent celiac disease, such a finding is not pathognomonic for the disease [59,61]. In the small intestine, the gluten-dependent autoantibodies target extracellular TG2 and may be detected as ***IgA deposits*** in biopsy tissues at the latent disease stage [62,63,64] (Figure 2). In fact, the IgA deposits in the duodenal biopsies accurately predicted forthcoming celiac disease better than IELs, γδ^+^ IELs, or serum autoantibodies [59]. The detection of intestinal TG2-abs by phage-antibody libraries is another possibility for diagnosis [52]. However, intestinal TG2-ab production is not only found in celiac disease [65]. Again, when finding an increased density of γδ^+^ IELs or IgA deposits in a patient with normal small intestinal mucosal morphology, it is recommended to check whether the patient belongs to the “celiac family” (i.e., are carrying either the HLA DQ2 or DQ8 molecules).

## 4. Extraintestinal Manifestations

### 4.1. Dermatitis Herpetiformis

In dermatitis herpetiformis, a gluten-induced and gluten-dependent manifestation occurs outside the gut even in the absence of intestinal mucosal villous atrophy [8,66]. Today, up to 30% of patients with dermatitis herpetiformis have a normal small intestinal mucosal lining [67]. Typically, the disease manifests with itchy papules and vesicles on the elbows, knees and buttocks, and overt gastrointestinal symptoms are rare [67,68]. When patients without enteropathy are challenged with extra gluten, their small intestinal mucosae deteriorate in a way typical of celiac disease [38,39,40]. When no enteropathy is present, patients only test positively for serum TG2-abs and endomysial antibodies in 40% of cases [67]. However, the patients could be serum endomysial autoantibody positive already while having normal small intestinal mucosa prior to any evidence of skin eruptions [44]. If searched for, the autoimmune effect to the morphologically normal intestinal mucosa caused by an environmental trigger—the daily ingestion of gluten—is, in fact, present. Mucosal inflammatory markers (i.e., the high density of γδ^+^ IELs) shows this [35]. Furthermore, in patients who are negative for serum autoantibodies, the antibodies are found at the mucosal level targeting extracellular TG2 [62,69,70], which is a finding typical for an existing disease that is not manifest at the mucosal architectural level (Figure 2).

We infer that patients with gluten-triggered extra-intestinal manifestations, who are now classified as having dermatitis herpetiformis but show a normal small intestinal mucosa, do not belong to the category of potential celiac disease. These patients may even be suffering from osteoporosis and experience bone fractures. Clearly, it is a treatable disease [67,68,71]. In the following, we use parallel reasoning for patients having other gluten-dependent extra-intestinal manifestations occurring at the latent stage of celiac disease.

### 4.2. Central and Peripheral Nervous System

Gluten-induced neurological manifestations including gluten ataxia are common in adult celiac disease [19,20,21,22] and occur in children [2]. Hadjivassiliou et al. noticed that gluten sensitivity was found in patients with neurological disease, and they screened the patients with gliadin antibodies [72]. They also showed that neurological complications occurred during the latent stage of celiac disease. Their use of gliadin antibodies created some skepticism toward the findings, but today gluten ataxia has become a gluten-induced entity in itself, similar to dermatitis herpetiformis [20,73]. In fact, it was shown that gluten ataxia might respond to a strict gluten-free diet even in the absence of an enteropathy [74,75]. Serum transglutaminase 6 antibodies are used for detecting gluten ataxia in patients with and without small intestinal mucosal lesions. Negative seroconversion results from a gluten-free diet [75]. Further evidence that gluten ataxia without enteropathy belongs to the celiac spectrum comes from the finding that TG2-specific autoantibody deposits were detected in the intestinal mucosa [74]. The patients were HLA DQ2-positive or DQ8-positive. All of the control ataxia patients were negative for celiac-type HLA, and they had no IgA deposits in the mucosa [74]. In one gluten ataxia patient, similar TG2-targeted IgA deposits were found in the small vessels of the brain [74]. In a different cohort of idiopathic ataxia patients, the TG2-targeted IgA deposits were again detected, even in the absence of circulating TG2-abs [76].

Gluten-induced peripheral nervous system involvement often expresses as a symmetrical sensorimotor axonal peripheral neuropathy [77]. In patients with or without enteropathy, neuropathies cannot be differentiated based on clinical, genetic, or immunological grounds [78,79].

### 4.3. Bone Disease

Bone diseases, osteopenia, osteoporosis, and even fractures are highly prevalent in untreated celiac disease [14,80,81]. Strict gluten-free diets improve bone health in celiac disease and are an effective therapy for long-term bone mineral recovery [13,82].

Latent celiac disease patients, before manifesting an overt disease, might suffer from gluten-dependent symptoms as well as osteopenia and osteoporosis [62,83,84,85,86]. Kaukinen et al. showed that eight of 10 patients without villous atrophy had a bone disease, and they all were DQ2 positive [83]. On biopsy, it was shown that they belonged to the celiac spectrum since they had increased densities of γδ^+^ IELs. Furthermore, their initial TG2 and endomysial antibodies normalized with a gluten-free diet. Dickey et al. again measured bone mineral density in 31 endomysial antibody-positive patients who were excluded for celiac disease (i.e., classified as having Marsh 0 or Marsh 1 lesions). They found osteopenia to be present in 30% and osteoporosis in 10% of these patients, and the degree of bone disease did not differ from that found in patients diagnosed with overt celiac disease. Negative seroconversion followed upon implementation of a gluten-free diet in the 26 of the 27 patients with normal biopsies. On the contrary, eight patients continued their gluten-containing diet, and seven of them evolved toward villous atrophy compatible with celiac disease within one to two years [84]. Kurppa et al. proved that the gluten-free diet had a positive effect on the bone mineral density in endomysial antibody-positive patients with normal villous morphology, which is similar to those with celiac-type enteropathy [85]. Zanini et al. concluded that celiac disease patients with mild enteropathy have various markers of existing malabsorption including bone disease, and, thus, require treatment with a gluten-free diet [86].

Patients with true potential celiac disease are also a risk of fractures. Pasternack et al. showed that dermatitis herpetiformis patients reported earlier fractures in 45 out of 222 cases at diagnosis. Altogether, 16% of the fractures had occurred in patients with normal small intestinal histology, 35% occurred in patients with partial villous atrophy, and 49% occurred in patients with subtotal villous atrophy in Reference [71].

### 4.4. Liver Diseases

Celiac disease may initially present as a monosymptomatic liver disease, such as cryptogenic hypertransaminasaemia or autoimmunue-type of liver damage [23,24,25,87]. There are few reports of liver injury in potential or latent celiac disease. Zanini et al. observed that celiac disease with mild enteropathy and positive celiac disease-related serology is not a mild disease. Moreover, they showed alanine aminotransferase serum values to be elevated in 9/121 (8%), γ-glutamyltransferase in 5/102 (5%), and alkaline phosphatase in 6/101 (6%) of patients. The authors concluded that these patients should also be treated [86].

### 4.5. Other Extraintestinal Manifestations

#### 4.5.1. Permanent Tooth Dental Enamel Defects

Adult patients with celiac disease and dermatitis herpetiformis, as well as children with dermatitis herpetiformis, show celiac-type dental enamel defects in their permanent dentition [15,88,89,90]. Typical enamel defects were found in all healthy family members of celiac disease patients found to have manifest mucosal lesions. Furthermore, these celiac-type enamel defects occurred without small intestinal changes and were strongly associated with the HLA DR3 [34]. Importantly, these permanent tooth enamel defects are induced by gluten ingestion in early childhood, when the enamel is developing (i.e., at the latent stage of celiac disease and dermatitis herpetiformis).

#### 4.5.2. Malignancies

In 1986, Freeman and Chiu reported that intestinal lymphoma might appear at the latent stage of celiac disease when the mucosa is morphologically normal [33]. However, it is not known whether untreated patients without a manifest mucosal lesion carry an increased risk of malignancy. However, there are many complications, including malignancies, that may occur in adulthood when the patient is undiagnosed by ingesting gluten. Celiac disease patients diagnosed at an adult and elderly age have not had manifest mucosal lesions from early childhood (Figure 1) [42,43,44,45,48,49].

**Figure 3:**
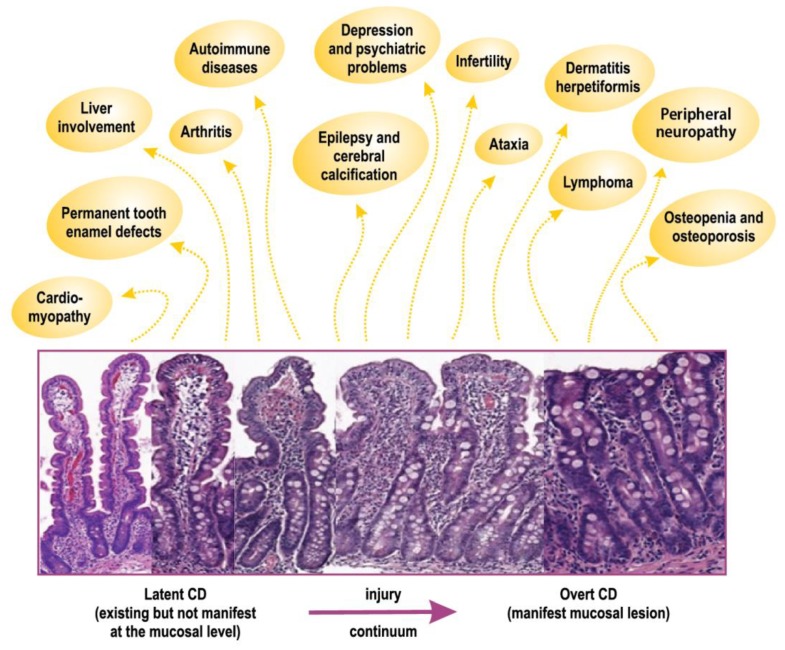
Celiac trait. Gluten-induced extra-intestinal manifestations exist in both patients with normal (latent celiac disease, CD) and diseased small intestinal mucosa (overt CD). Drawing adapted from the “cooking pot” of splashing extra-intestinal manifestations, which is first presented at the International Celiac Disease Symposium in Dublin 1992, and from drawings in references No. 34, 53, and 54.

Figure 3 indicates that all the extra-intestinal manifestations induced by gluten in untreated celiac disease can be detected in the latent stage of the disease.

## 5. Celiac Trait

Celiac disease diagnosis requires a gluten-induced small intestinal mucosal lesion. As indicated in Figure 3, the so-called “flat” lesion is the end stage of the mucosal injury. Figure 3 also summarizes our review and shows that, when there is a gluten-induced and gluten-dependent extra-intestinal manifestation in celiac disease, the manifestation can be found in patients before the mucosa is diseased or when it shows only minor non-specific changes. For susceptible persons, upon gluten ingestion, celiac disease develops gradually from normal mucosal morphology through mucosal inflammation, crypt hyperplasia, and villous atrophy to the “flat” mucosal lesions [91]. When the mucosa is morphologically normal and, for example, bones are already fracturing due to gluten ingestion, we should not call this condition potential celiac disease [38]. Moreover, we should not wait for the manifest mucosal lesion to develop (i.e., celiac disease). The patient deserves accurate treatment early. The benefit of treating these patients may be due to correction of micronutrient deficiencies having an impact on extra-intestinal manifestations [58]. We suggest that the term celiac trait be used in these cases [1,32,51].

## References

[1] Mäki M., Collin P. (1997). Celiac disease. Lancet.

[2] Laurikka P., Nurminen S., Kivelä L., Kurppa K. (2018). Extraintestinal manifestations of celiac disease: Early detection for better long-term outcomes. Nutrients.

[3] Pinto-Sanchez M.I., Bercik P., Verdu E.F., Bai J.C. (2015). Extraintestinal manifestations of celiac disease. Dig. Dis..

[4] Kaukinen K., Collin P., Mäki M. (2010). Celiac disease-a diagnostic and therapeutic challenge. Duodecim.

[5] Collin P., Reunala T., Rasmussen M., Kyrönpalo S., Pehkonen E., Laippala P., Mäki M. (1997). High incidence and prevalence of adult celiac disease: Augmented diagnostic approach. Scand. J. Gastroenterol..

[6] Lohi S., Mustalahti K., Kaukinen K., Laurila K., Collin P., Rissanen H., Lohi O., Bravi E., Gasparin M., Reunanen A. (2007). Increasing prevalence of celiac disease over time. Aliment. Pharmacol. Ther..

[7] Virta L.J., Kaukinen K., Collin P. (2009). Incidence and prevalence of diagnosed celiac disease in Finland: Results of effective case finding in adults. Scand. J. Gastroenterol..

[8] Fry L., Riches D.J., Seah P.P., Hoffbrand A.V. (1973). Clearance of skin lesions in dermatitis herpetiformis after gluten withdrawal. Lancet.

[9] Reunala T., Kosnai I., Karpati S., Kuitunen P., Török E., Savilahti E. (1984). Dermatitis herpetiformis: Jejunal findings and skin response to gluten-free diet. Arch. Dis. Child..

[10] Reunala T., Mäki M. (1993). Dermatitis herpetiformis: A genetic disease. Eur. J. Dermatol..

[11] Hervonen K., Karell K., Holopainen P., Collin P., Partanen J., Reunala T. (2000). Concordance of dermatitis herpetiformis and celiac disease in monozygous twins. J. Investig. Dermatol..

[12] Molteni N., Caraceni M.P., Bardella M.T., Ortolani S., Gandolini G.G., Bianchi P. (1990). Bone mineral density in adult celiac patients and the effect of gluten-free diet from childhood. Am. J. Gastroenterol..

[13] Mustalahti K., Collin P., Sievänen H., Salmi J., Mäki M. (1999). Osteopenia in patients with clinically silent celiac disease warrants screening. Lancet.

[14] Heikkilä K., Pearce J., Mäki M., Kaukinen K. (2015). Celiac disease and bone fractures: A systematic review and meta-analysis. J. Clin. Endocrinol. Metab..

[15] Trotta L., Biagi F., Bianchi P.I., Marchese A., Vattiato C., Balduzzi D., Collesano V., Corazza G.R. (2013). Dental enamel defects in adult celiac disease: Prevalence and correlation with symptoms and age at diagnosis. Eur. J. Intern. Med..

[16] Mäki M., Hällström O., Verronen P., Reunala T., Lahdeaho M.L., Holm K., Visakorpi J.K. (1988). Reticulin antibody, arthritis, and celiac disease in children. Lancet.

[17] Collin P., Korpela M., Hällström O., Viander M., Keyriläinen O., Mäki M. (1992). Rheumatic complaints as a presenting symptom in patients with celiac disease. Scand. J. Rheumatol..

[18] Daron C., Soubrier M., Mathieu S. (2017). Occurrence of rheumatic symptoms in celiac disease: A meta-analysis: Comment on the article “Osteoarticular manifestations of celiac disease and non-celiac gluten hypersensitivity” by Dos Santos and Lioté. Jt. Bone Spine.

[19] Gobbi G., Bouquet F., Greco L., Lambertini A., Tassinari C.A., Ventura A., Zaniboni M.G. (1992). Celiac disease, epilepsy and cerebral calcifications. The Italian Working Group on Celiac Disease and Epilepsy. Lancet.

[20] Hadjivassiliou M., Grunewald R.A., Chattopadhyay A.K., Davies-Jones G.A., Gibson A., Jarrat J.A., Kandler R.H., Lobo A., Powell T., Smith C.M. (1998). Clinical, radiological, neurophysiological, and neuropathological characteristics of gluten ataxia. Lancet.

[21] Luostarinen L., Pirttilä T., Collin P. (1999). Celiac disease presenting with neurological disorders. Eur. Neurol..

[22] Zis P., Sarrigiannis P.G., Rao D.G., Hadjivassiliou M. (2018). Gluten neuropathy: Prevalence of neuropathic pain and the role of gluten-free diet. J. Neurol..

[23] Volta U., De Franceschi L., Lari F., Molinaro N., Zoli M., Bianchi F.B. (1998). Celiac disease hidden by cryptogenic hypertransaminasaemia. Lancet.

[24] Kaukinen K., Halme L., Collin P., Färkkilä M., Mäki M., Vehmanen P., Partanen J., Höckerstedt K. (2002). Celiac disease in patients with severe liver disease: Gluten-free diet may reverse hepatic failure. Gastroenterology.

[25] Korpimäki S., Kaukinen K., Collin P., Haapala A.-M., Holm P., Laurila K., Kurppa K., Saavalainen P., Haimila K., Partanen J. (2011). Gluten-sensitive hypertransaminasemia in celiac disease: An infrequent and often subclinical finding. Am. J. Gastroenterol..

[26] Sher K.S., Jayanthi V., Probert C.S., Stewart C.R., Mayberry J.F. (1994). Infertility, obstetric and gynaecological problems in celiac sprue. Dig. Dis..

[27] Tersigni C., Castellani R., de Waure C., Fattorossi A., De Spirito M., Gasbarrini A., Scambia G., Di Simone N. (2014). Celiac disease and reproductive gdisorders: Meta-analysis of epidemiologic associations and potential pathogenic mechanisms. Hum. Reprod. Update.

[28] Ventura A., Magazzu G., Greco L. (1999). Duration of exposure to gluten and risk for autoimmune disorders in patients with celiac disease. Gastroenterology.

[29] Cosnes J., Cellier C., Viola S., Colombel J.F., Michaud L., Sarles J., Hugot J.P., Ginies J.L., Dabadie A., Mouterde O. (2008). Groupe D’Etude et de Recherche Sur la Maladie Coeliaque. Incidence of autoimmune diseases in celiac disease: Protective effect of the gluten-free diet. Clin. Gastroenterol. Hepatol..

[30] Holmes G.K.T., Prior P., Lane M.R., Pope D., Allan R.N. (1989). Malignancy in celiac disease—Effect of a gluten free diet. Gut.

[31] Tio M., Cox M.R., Eslick G.D. (2012). Meta-analysis: Celiac disease and the risk of all-cause mortality, any malignancy and lymphoid malignancy. Aliment. Pharmacol. Ther..

[32] Mäki M., Delvin E.E., Lentze M.J. (2001). Celiac disease. Gastrointestinal Functions.

[33] Freeman H.J., Chiu B.K. (1986). Multifocal small bowel lymphoma and latent celiac sprue. Gastroenterology.

[34] Mäki M., Aine L., Lipsanen V., Koskimies S. (1991). Dental enamel defects in first-degree relatives of celiac disease patients. Lancet.

[35] Savilahti E., Reunala T., Mäki M. (1992). Increase of lymphocytes bearing the gamma/delta T-cell receptor in the jejunum of patients with dermatitis herpetiformis. Gut.

[36] Mäki M., Huupponen T., Holm K., Hällström O. (1995). Seroconversion of reticulin autoantibodies predicts celiac disease in insulin-dependent diabetes mellitus. Gut.

[37] Ludvigsson J.F., Leffler D.A., Bai J.C., Biagi F., Fasano A., Green P.H., Hadjivassiliou M., Kaukinen K., Kelly C.P., Leonard J.N. (2013). The Oslo definitions for celiac disease and related terms. Gut.

[38] Weinstein W.M. (1974). Latent celiac sprue. Gastroenterology.

[39] Ferguson A., Blackwell J.N., Barnetson R.S. (1987). Effects of additional dietary gluten on the small-intestinal mucosa of volunteers and of patients with dermatitis herpetiformis. Scand. J. Gastroenterol..

[40] Chorzelski T.P., Rosinska D., Beutner E.H., Sulej J., Kumar V. (1988). Aggressive gluten challenge of dermatitis herpetiformis cases converts them from seronegative to seropositive for IgA-class endomysial antibodies. J. Am. Acad. Dermatol..

[41] Doherty M., Barry R.E. (1981). Gluten-induced mucosal changes in subjects without overt small-bowel disease. Lancet.

[42] Mäki M., Holm K., Koskimies S., Hallstrom O., Visakorpi J.K. (1990). Normal small bowel biopsy followed by celiac disease. Arch. Dis. Child..

[43] Mäki M., Holm K., Collin P., Savilahti E. (1991). Increase in γ/δ T cell receptor bearing lymphocytes in normal small bowel mucosa in latent celiac disease. Gut.

[44] Mäki M., Holm K., Lipsanen V., Hällström O., Viander M., Collin P., Savilahti E., Koskimies S. (1991). Serological markers and HLA genes among healthy first-degree relatives of patients with celiac disease. Lancet.

[45] Collin P., Helin H., Mäki M., Hällström O., Karvonen A.L. (1993). Follow-up of patients positive in reticulin and gliadin antibody tests with normal small-bowel biopsy findings. Scand. J. Gastroenterol..

[46] Ferguson A., Arranz E., O’Mahony S. (1993). Clinical and pathological spectrum of celiac disease—Active, silent, latent, potential. Gut.

[47] Troncone R. (1995). Latent celiac disease in Italy. Acta Paediatr..

[48] Corazza G.R., Andreani M.L., Biagi F., Bonvicini F., Bernardi M., Gasbarrini G. (1996). Clinical, pathological, and antibody pattern of latent celiac disease: Report of three adult cases. Am. J. Gastroenterol..

[49] Vilppula A., Kaukinen K., Luostarinen L., Kerkelä I., Patrikainen H., Valve T., Mäki M., Collin P. (2009). Increasing prevalence and high incidence of celiac disease in elderly people: A population-based study. BMC Gastroenterol..

[50] Kaukinen K., Collin P., Mäki M. (2007). Latent celiac disease or celiac disease beyond villous atrophy?. Gut.

[51] Mäki M. (2012). Lack of consensus regarding definitions of celiac disease. Nat. Rev. Gastroenterol. Hepatol..

[52] Not T., Ziberna F., Vatta S., Quaglia S., Martelossi S., Villanacci V., Marzari R., Florian F., Vecchiet M., Sulic A.M. (2011). Cryptic genetic gluten intolerance revealed by intestinal antitransglutaminase antibodies and response to gluten-free diet. Gut.

[53] Kurppa K., Collin P., Viljamaa M., Haimila K., Saavalainen P., Partanen J., Laurila K., Huhtala H., Paasikivi K. (2009). Diagnosing mild enteropathy celiac disease: A randomized, controlled clinical study. Gastroenterology.

[54] Husby S., Koletzko S., Korponay-Szabó I.R., Mearin M.L., Phillips A., Shamir R., Troncone R., Giersiepen K., Branski D., Catassi C. (2012). European Society for Pediatric Gastroenterology, Hepatology, and Nutrition Guidelines for the Diagnosis of Celiac Disease. J. Pediatr. Gastroenterol. Nutr..

[55] Mäki M. (1995). The humoral immune system in celiac disease. Baillière’s Clin. Gastroenterol..

[56] Simon-Vecsei Z., Király R., Bagossi P., Tóth B., Dahlbom I., Caja S., Csősz E., Lindfors K., Sblattero D., Nemes E. (2012). A single conformational transglutaminase 2 epitope contributed by three domains is critical for celiac antibody binding and effects. Proc. Natl. Acad. Sci. USA.

[57] Yu X.B., Uhde M., Green P.H., Alaedini A. (2018). Autoantibodies in the extraintestinal manifestations of celiac disease. Nutrients.

[58] Rostami K., Aldulaimi D., Holmes G., Johnson M.W., Robert M., Srivastava A., Fléjou J.-F., Sanders D.S., Volta U., Derakhshan M.H. (2015). Microscopic enteritis: Bucharest consensus. World J. Gastroenterol..

[59] Salmi T.T., Collin P., Järvinen O., Haimila K., Partanen J., Laurila K., Korponay-Szabo I.R., Huhtala H., Reunala T., Mäki M. (2006). Immunoglobulin A autoantibodies against transglutaminase 2 in the small intestinal mucosa predict forthcoming celiac disease. Aliment. Pharmacol. Ther..

[60] Holm K., Mäki M., Savilahti E., Lipsanen V., Laippala P., Koskimies S. (1992). Intraepithelial gamma/delta T-cell-receptor lymphocytes and genetic susceptibility to celiac disease. Lancet.

[61] Iltanen S., Holm K., Partanen J., Laippala P., Mäki M. (1999). Increased density of jejunal γδ+ T cells in patients having normal mucosa—Marker of operative autoimmune mechanisms?. Autoimmunity.

[62] Korponay-Szabo I.R., Halttunen T., Szalai Z., Laurila K., Király R., Kovács J.B., Fésüs L., Mäki M. (2004). In vivo targeting of intestinal and extraintestinal transglutaminase 2 by celiac autoantibodies. Gut.

[63] Kaukinen K., Peräaho M., Collin P., Partanen J., Woolley N., Kaartinen T., Nuutinen T., Halttunen T., Mäki M., Korponay-Szabo I. (2005). Small-bowel mucosal transglutaminase 2-specific IgA deposits in celiac disease without villous atrophy: A prospective and randomized clinical study. Scand. J. Gastroenterol..

[64] Koskinen O., Collin P., Korponay-Szabo I., Salmi T., Iltanen S., Haimila K., Partanen J., Mäki M., Kaukinen K. (2008). Gluten-dependent small bowel mucosal transglutaminase 2-specific IgA deposits in overt and mild enteropathy celiac disease. J. Pediatr. Gastroenterol. Nutr..

[65] Maglio M., Ziberna F., Aitoro R., Discepolo V., Lania G., Bassi V., Miele E., Not T., Troncone R., Auricchio R. (2017). Intestinal production of anti-tissue transglutaminase 2 antibodies in patients with diagnosis other than celiac disease. Nutrients.

[66] Reunala T., Blomqvist K., Tarpila S., Halme H., Kangas K. (1977). Gluten-free diet in dermatitis herpetiformis. I. Clinical response of skin lesions in 81 patients. Br. J. Dermatol..

[67] Mansikka E., Hervonen K., Kaukinen K., Collin P., Huhtala H., Reunala T., Salmi T. (2018). Prognosis of dermatitis herpetiformis patients with and without villous atrophy at diagnosis. Nutrients.

[68] Reunala T., Salmi T.T., Hervonen K., Kaukinen K., Collin P. (2018). Dermatitis herpetiformis: A common extraintestinal manifestation of celiac disease. Nutrients.

[69] Salmi T.T., Hervonen K., Laurila K., Collin P., Mäki M., Koskinen O., Huhtala H., Kaukinen K., Reunala T. (2014). Small bowel transglutaminase 2-specific IgA deposits in dermatitis herpetiformis. Acta Derm. Venereol..

[70] Salmi T., Collin P., Korponay-Szabo I.R., Laurila K., Partanen J., Huhtala H., Kiraly R., Lorand L., Reunala T., Mäki M. (2006). Endomysial antibody-negative celiac disease: Clinical characteristics and intestinal autoantibody deposits. Gut.

[71] Pasternack C., Mansikka E., Kaukinen K., Hervonen K., Reunala T., Collin P., Huhtala H., Mattila V.M., Salmi T. (2018). Self-reported fractures in dermatitis herpetiformis compared to celiac disease. Nutrients.

[72] Hadjivassiliou M., Gibson A., Davies-Jones G.A., Lobo A.J., Stephenson T.J., Milford-Ward A. (1996). Does cryptic gluten sensitivity play a part in neurological illness?. Lancet.

[73] Hadjivassiliou M., Grünewald R.A., Sanders D.S., Zis P., Croall I., Shanmugarajah P.D., Sarrigiannis P.G., Trott N., Wild G., Hoggard N. (2018). The significance of low titre antigliadin antibodies in the diagnosis of gluten ataxia. Nutrients.

[74] Hadjivassiliou M., Davies-Jones G.A.B., Sanders D.S., Grunewald R.A. (2003). Dietary treatment of gluten ataxia. J. Neurol. Neurosurg. Psychiatry.

[75] Hadjivassiliou M., Mäki M., Sanders D.S., Williamson C.A., Grunewald R.A., Woodroofe N.M., Korponay-Szabo I.R. (2006). Antibody targeting of brain and intestinal trasnglutaminase in gluten ataxia. Neurology.

[76] Hadjivassiliou M., Aeschlimann P., Sanders D.S., Mäki M., Kaukinen K., Grünewald R.A., Bandmann O., Woodroofe N., Haddock G., Aeschlimann D.P. (2013). Transglutaminase 6 antibodies in the diagnosis of gluten ataxia. Neurology.

[77] Zis P., Sarrigiannis P.G., Rao D.G., Hadjivassiliou M. (2018). Quality of life in patients with gluten neuropathy: A case-controlled study. Nutrients.

[78] Hadjivassiliou M., Grünewald R.A., Kandler R.H., Chattopadhyay A.K., Jarratt J.A., Sanders D.S., Sharrack B., Wharton S.B., Davies-Jones G.A. (2006). Neuropathy associated with gluten sensitivity. J. Neurol. Neurosurg. Psychiatry.

[79] Hadjivassiliou M., Kandler R.H., Chattopadhyay A.K., Davies-Jones A.G., Jarratt J.A., Sanders D.S., Sharrack B., Grünewald R.A. (2006). Dietary treatment of gluten neuropathy. Muscle Nerve.

[80] Larussa T., Suraci E., Nazionale I., Abenavoli L., Imeneo M., Luzza F. (2012). Bone mineralization in celiac disease. Gastroenterol. Res. Pract..

[81] Zanchetta M.B., Longobardi V., Bai J.C. (2016). Bone and celiac disease. Curr. Osteoporos. Rep..

[82] Grace-Farfaglia P. (2015). Bones of contention: Bone mineral density recovery in celiac disease—A systematic review. Nutrients.

[83] Kaukinen K., Mäki M., Partanen J., Sievänen H., Collin P. (2001). Celiac disease without villous atrophy: Revision of criteria called for. Dig. Dis. Sci..

[84] Dickey W., Hughes D.F., McMillan S.A. (2005). Patients with serum IgA endomysial antibodies and intact duodenal villi: Clinical characteristics and management options. Scand. J. Gastroenterol..

[85] Kurppa K., Collin P., Sievänen H., Huhtala H., Mäki M., Kaukinen K. (2010). Gastrointestinal symptoms, quality of life and bone mineral density in mild enteropathic celiac disease: A prospective clinical trial. Scand. J. Gastroenterol..

[86] Zanini B., Caselani F., Magni A., Turini D., Ferraresi A., Lanzarotto F., Villanacci V., Carabellese N., Ricci C., Lanzini A. (2013). Celiac disease with mild enteropathy is not mild disease. Clin. Gastroenterol. Hepatol..

[87] Volta U. (2009). Pathogenesis and clinical significance of liver injury in celiac disease. Clin. Rev. Allergy Immunol..

[88] Aine L., Mäki M., Collin P., Keyriläinen O. (1990). Dental enamel defects in celiac disease. J. Oral Pathol. Med..

[89] Aine L., Reunala T., Mäki M. (1991). Dental enamel defects in children with dermatitis herpetiformis. J. Pediatr..

[90] Aine L., Mäki M., Reunala T. (1992). Celiac-type dental enamel defects in patients with dermatitis herpetiformis. Acta Derm. Venereol..

[91] Marsh M.N. (1992). Gluten, major histocompatibility complex, and the small intestine. A molecular and immunologic approach to the spectrum of gluten sensitivity (‘celiac sprue’). Gastroenterology.

